# Draft genome of the sea cucumber *Apostichopus japonicus* and genetic polymorphism among color variants

**DOI:** 10.1093/gigascience/giw006

**Published:** 2017-01-07

**Authors:** Jihoon Jo, Jooseong Oh, Hyun-Gwan Lee, Hyun-Hee Hong, Sung-Gwon Lee, Seongmin Cheon, Elizabeth M A Kern, Soyeong Jin, Sung-Jin Cho, Joong-Ki Park, Chungoo Park

**Affiliations:** 1 School of Biological Sciences and Technology, Chonnam National University, Gwangju 61186, Republic of Korea; 2 Marine Ecological Disturbing and Harmful Organisms Research Center, Department of Oceanography, Chonnam National University, Gwangju 61186, Republic of Korea; 3 Division of EcoScience, Ewha Womans University, Seoul 03760, Republic of Korea; 4 Department of Biology, College of Natural Sciences, Chungbuk National University, Cheongju, Chungbuk 28644, Republic of Korea

**Keywords:** Sea cucumber genome, *Apostichopus japonicas*, Color variants, Genetic variation, Population genomics

## Abstract

The Japanese sea cucumber (*Apostichopus japonicus* Selenka 1867) is an economically important species as a source of seafood and ingredient in traditional medicine. It is mainly found off the coasts of northeast Asia. Recently, substantial exploitation and widespread biotic diseases in *A. japonicus* have generated increasing conservation concern. However, the genomic knowledge base and resources available for researchers to use in managing this natural resource and to establish genetically based breeding systems for sea cucumber aquaculture are still in a nascent stage. A total of 312 Gb of raw sequences were generated using the Illumina HiSeq 2000 platform and assembled to a final size of 0.66 Gb, which is about 80.5% of the estimated genome size (0.82 Gb). We observed nucleotide-level heterozygosity within the assembled genome to be 0.986%. The resulting draft genome assembly comprising 132 607 scaffolds with an N50 value of 10.5 kb contains a total of 21 771 predicted protein-coding genes. We identified 6.6–14.5 million heterozygous single nucleotide polymorphisms in the assembled genome of the three natural color variants (green, red, and black), resulting in an estimated nucleotide diversity of 0.00146. We report the first draft genome of *A. japonicus* and provide a general overview of the genetic variation in the three major color variants of *A. japonicus*. These data will help provide a comprehensive view of the genetic, physiological, and evolutionary relationships among color variants in *A. japonicus*, and will be invaluable resources for sea cucumber genomic research.

## Data description

### Background information on *Apostichopus japonicus*

**Figure 1.**
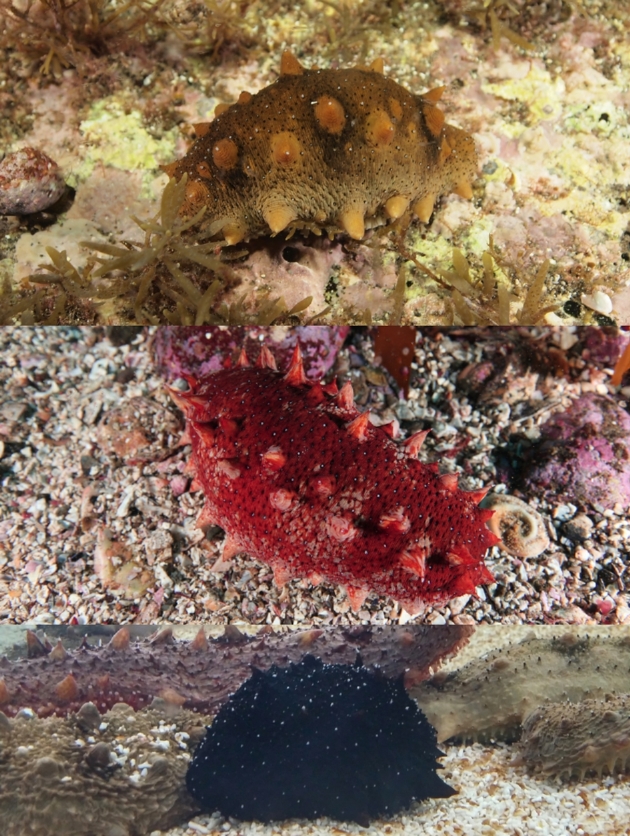
Three color variants of *A. japonicus* (green, red, and black).

The class Holothuroidea (also known as sea cucumbers) belongs to the phylum Echinodermata and comprises approximately 1250 recorded species worldwide, including some species that are of commercial and medical value [1, 2]. *Apostichopus japonicus* Selenka 1867 is one of the well-known, commercially important sea cucumber species and occurs in the northwestern Pacific coast including China, Japan, Korea, and the Far Eastern seas. This species exhibits a wide array of dorsal/ventral color variants (in particular green, red, and black; Fig. 1), which differ in their biological and morphological attributes (e.g., shape of ossicle, habitat preference, spawning period, and polian vesicles) [1, 3]. The red variant is found on rock pebbles and gravel substrate and has higher salinity and temperature tolerance than the other color variants [4, 5]. Green and black variants are found on sandy and muddy bottoms at shallower depths, and the green variant has greater plasticity in thermotolerance than the red variant [6, 7].

Recently, overexploitation and the prevalence of biotic diseases (viral infections) in sea cucumber aquaculture have generated increasing conservation concern [8, 9]. However, the genomic knowledge base and resources available for researchers to use in managing this natural resource or establishing genetically based breeding systems are still in a nascent stage [10].

### Sample collection and genomic DNA extraction

Specimens of the three color *A. japonicus* variants (green, red, and black) were collected from same geographical location (GPS data: 34.1 N, 127.18 E, Geomun-do, Yeosu, Republic of Korea). Genomic DNA of each color variant was extracted manually from body wall tissues of single male specimens. Briefly, we ground the tissues to fine powder using mortar and pestle with liquid nitrogen freezing. Tissue powders were digested for 1 hour at 65°C in CTAB buffer (2% cetyltrimethylammonium bromide, 1.4 M NaCl, 20 mM EDTA, 100 mM Tris-HCl, and pH 8.0), followed by Phenol/Chloroform extraction and ethanol precipitation.

### Sequencing and quality control

**Figure 2.**
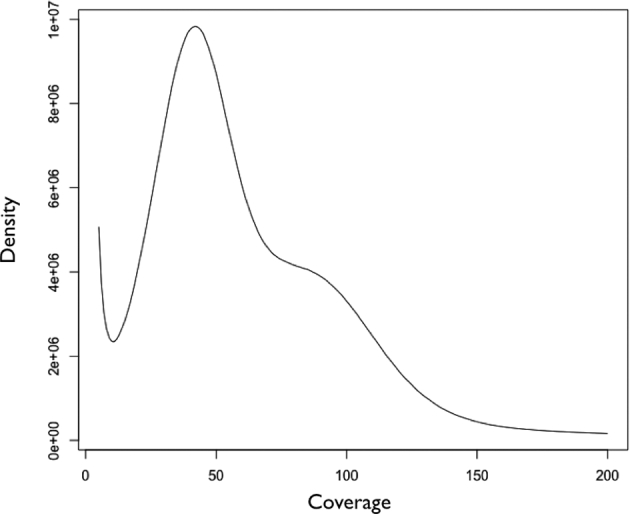
K-mer distribution of the *A. japonicus* genome.

Using the standard protocol provided by Illumina (San Diego, USA), we constructed both short-insert (180 and 400 bp) and long-insert (2 kb) libraries for 2 × 101 bp paired-end reads, which were sequenced using a HiSeq 2000 instrument. For the green color variant, a total of 225 Gb of raw data was generated from all three libraries. In the case of the red and black color variants, 40 and 47 Gb of raw reads, respectively, were produced by 400 bp short-insert library. The raw reads were preprocessed using Trimmomatic v0.33 [11] and Trim Galore [12], in which reads containing adapter sequences, poly-N sequences, or low-quality bases (below a mean Phred score of 20) were removed. To correct errors in the raw sequences, we used ALLPATHS-LG v52488 [13]. Approximately 208, 39, and 42 billion clean reads were obtained for green, red, and black color variant samples, respectively (Table 1). The *A. japonicus* genome size was estimated to be approximately 0.9 Gb based on k-mer measurement (Fig. 2), which is fully consistent with genome size measured by flow cytometry (∼0.82 Gb) [14]. Based on this estimation, the clean sequence reads correspond to about 356-fold coverage of the *A. japonicus* genome.

**Figure 3.**
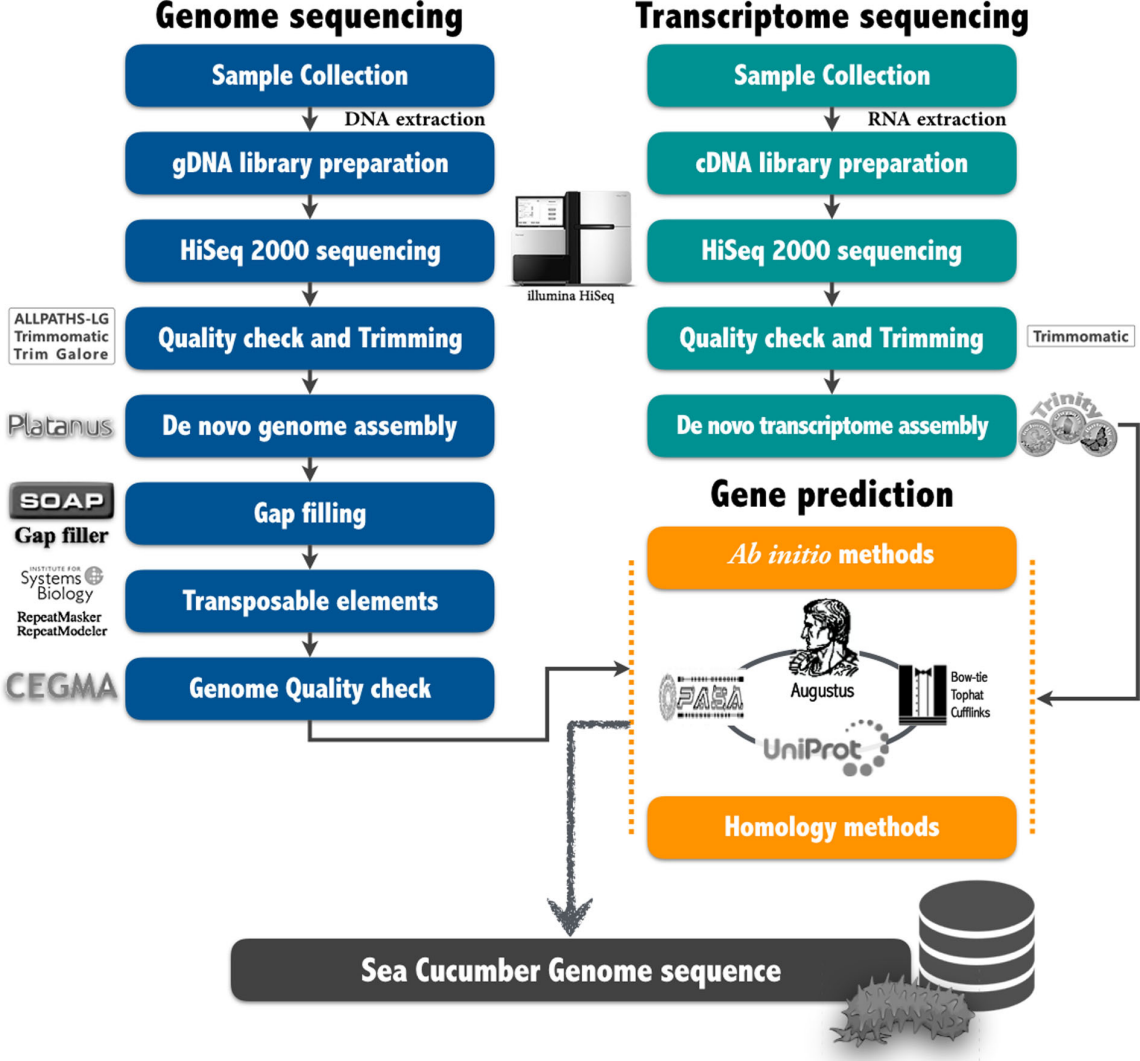
Schematic workflow of *A. japonicus* genome assembly and annotation. The left side represents the genome assembly and the right side represents the transcriptome assembly that was performed in previous publications. To achieve suitable gene prediction, we integrated these two assembly results.

**Table 1.** Statistics on total reads of the *A. japonicus* genome

Variants	Insertion size (bp)	Total reads^a^ (raw data)	Total reads^a^ (w/o adaptor)	Total reads^a^ (error corrected)	% error corrected
Green	180	498 608 646	474 117 288	466 062 920	1.70
	400	897 432 174	842 766 704	831 964 242	1.28
	2000 (v1)	293 701 464	270 513 434	268 573 812	0.72
	2000 (v2)	538 359 438	496 446 984	493 387 418	0.62
	Total	2228 101 722	2083 844 410	2059 988 392	1.14
Red	400	397 799 042	394 984 810	383 734 440	2.85
Black	400	460 597 940	423 543 558	416 007 614	1.78

^a^The length of each read is 101 bp.

### Assembly

For whole-genome assembly, we used reads only from green color variant libraries and employed Platanus v1.2.4 [15], which is well suited for high-throughput short reads and heterozygous diploid genomes. Briefly, error corrected paired-end (insert size: 180 bp and 400 bp) reads were input for contig assembly. Next, all cleaned paired-end (insert size: 180 bp and 400 bp) and mate-paired (insert size: two 2 kb samples) reads were mapped onto the contigs for scaffold building and were utilized for gap filling (any nucleotide represented by “N” in scaffolds). After gap filling by Platanus, the gaps that still remained in the resulting scaffolds were closed using GapCloser (a module of SOAPdenovo2 [16]). The final genome assembly was 0.66 Gb in total length, which is about 80.5% of the estimated genome size by flow cytometry (0.82 Gb) [14], and is composed of 132 607 scaffolds and unscaffolded contigs (that are longer than or equal to 1 kb) with an N50 value of 10.5 kb (Table 2). We assessed the completeness of the assembly using CEGMA v2.4.010312 [17] and BUSCO v1.22 [18]. Then 73.4% of the core eukaryotic genes (based on the 248 core essential genes) and 60.7% of the metazoan single-copy orthologs (based on the 843 genes) were identifiable in the genome. Because assembling highly heterozygous genomes is a major challenge in *de novo* genome sequencing, we further sought to explore whether there are other assemblers that could produce better genome assembly statistics. We applied two popular genome assemblers, SOAPdenovo2 2.04-r240 [16] and ALLPATHS-LG v52488 [13], and as expected [15], the Platanus assembler was superior to the others (Table S1).

**Table 2.** Statistics on *Apostichopus japonicus* genome assembly

Statistics	Values
Total assembled bases (bp)	664 375 288
Average length of scaffolds (bp)	5010
Number of scaffolds	132 607
Number of contigs	197 146
Length of longest scaffold (bp)	131 537
GC content (%)	35.92
Scaffold N50 (bp)	10 488
Contig N50 (bp)	5525
Number of genes	21 771
Number of exons per gene	4.67
Average exon length (bp)	209
Number of introns per gene	4.21
Average intron length (bp)	1048

### Annotation

To identify genomic repeat elements in the *A. japonicus* genome assembly, we ran RepeatMasker (version 4.0.6) [19] using the Repbase transposable element library (release 20150807) [20] and the *de novo* repeat library constructed by RepeatModeler (version 1.0.8) [21]. Approximately 27.2% of the *A. japonicus* genome was identified as interspersed repeats.

Protein-coding genes were predicted using four steps. First, *ab initio* gene prediction was performed with trained AUGUSTUS v3.2.1 [22] using hints from splicing alignment of transcripts to the repeat-masked assembled genome with BLAT [23] and PASA v2.0.2 [24]. To obtain high-quality spliced alignments of expressed transcript sequences for the AUGUSTUS training set, we collected high-throughput messenger RNA sequencing (RNA-seq) data from our previous [25] (from body wall tissue of adult stage specimens) and other transcriptome (from embryo, larva, and juvenile stages [developmental-stage specific]; from gonads, intestines, respiratory trees, and coelomic fluid of adults [tissue-specific]) [26] studies, and assembled reads from the RNA-seq dataset using Trinity v2.1.1 [27]. Second, for homology-based gene prediction, homologous proteins in other species (from UniProt [28]) were mapped to the repeat-masked assembled genome using tBLASTn [29] with an *E*-value ≤ 1 × 10^−5^. The aligned sequences were predicted using GeneWise v2.4.0 [30] to search for precise spliced alignment and gene structures. Third, for homology-based gene prediction with transcriptome evidence, existing RNA-seq reads [23, 25] were mapped to the repeat-masked assembled genome using TopHat v2.1.0 [31], and gene models were built using Cufflinks v2.2.1 [32]. Finally, the resulting gene sets from each approach were integrated into a comprehensive and non-redundant consensus gene set. We predicted a total of 21 771 (≥ 50 amino acids) genes in the assembled *A. japonicus* genome, including 101 776 exons (average 4.67 exons per gene), and an average gene size of 5402 nucleotides (average transcript size of 982 nucleotides) (Table. 2).

### Genetic polymorphism among natural color variants

To provide a general overview of the total genetic variation in the species, we realigned reads from the green color variant to the assembled genome using BWA v0.7.13 [33]. Picard v1.141 (http://broadinstitute.github.io/picard) was used to mark and remove duplicates. Before single nucleotide polymorphism (SNP) and small insertion and deletion (indel) calling, we realigned reads with indels using GATK RealignerTargetCreator and IndelRealigner v3.5 [34] to avoid misalignment around indels. Next, GATK Haplotypecaller was used to call SNPs and indels from the resulting sequences. In this study, we observed nucleotide-level heterozygosity within the assembled genome to be 0.986%; namely, we identified a total of 6 550 122 SNPs at the assembled genome, for a heterozygous SNP rate of 0.00986 per site. This high rate of nucleotide polymorphism is not uncommon in marine invertebrates and also has been found in the sea urchin genome (∼1%; at least one SNP per 100 bases) [35], which belongs to the same phylum.

To measure nucleotide diversity in *A. japonicus*, the aforementioned analyses were repeated for red and black color variants separately, and VCFtools v0.1.14 [36] with sliding window analysis (bin 10 kb, step 1 kb) was used to calculate nucleotide diversity. We identified 6.6–14.5 million heterozygous SNPs (1.7–3.7 million small indels) in the assembled genome from the three natural color variants (Table 3), resulting in an estimated nucleotide diversity of 0.00146.

**Table 3.** SNP and small indel statistics among three color variants

Variants	Percent heterozygous SNP loci	Percent small indel loci
Green	6 550 122	1 662 708
Red	14 509 713	3 681 007
Black	12 627 560	3 198 584

In summary, we report the first draft genome of *A. japonicus* Fig. 3 and provide a general overview of the genetic variation in its three color variants (green, red, and black). These data will help elucidate the genetic, physiological, and evolutionary relationships among different color variants in *A. japonicus* and will be invaluable resources for sea cucumber genomic research.

## Availability of supporting data

The raw dataset of all *A. japonicus* genome libraries and the assembly were deposited in the NCBI database with BioProject accession number PRJNA335936, SRA accession number SRP082485, and GenBank accession number MODV00000000. The additional dataset associated with genome annotation along with further supporting data are available in the GigaScience Database, GigaDB [37]. The RNA-seq datasets used in this study were downloaded from the ENA database with accession number PRJEB12167 and the NCBI database with SRA accession number SRA046386.

### Abbreviations

Indel: insertion and deletion; RNA-seq: high-throughput messenger RNA sequencing; SNP: single nucleotide polymorphism.

### Competing interests

The authors declare that they have no competing interests.

### Authors’ contributions

CP designed the study. CP, JKP, and SJC contributed to the project coordination. JJ, HGL, HHH, and SJ collected the samples and extracted the genomic DNA. CP, JO, SGL, and SC conducted the genome analyses. CP, JKP, JJ, and EK wrote the paper. All authors read and approved the final manuscript.

## Supplementary Material

GIGA-D-16-00085_Original_Submission.pdf

GIGA-D-16-00085_Revision_1.pdf

GIGA-D-16-00085_Revision_2.pdf

Response_to_Reveiwer_Comments_Original_Submission.pdf

Response_to_Reviewer_Comments_Revision_1.pdf

Reviewer_1_Report_(Original_Submission).pdf

Reviewer_2_Report_(Original_Submission).pdf

Table S1
