# Erratum to: Draft genome of the sea cucumber *Apostichopus japonicus* and genetic polymorphism among color variants

**DOI:** 10.1093/gigascience/gix069

**Published:** 2017-10-06

**Authors:** Jihoon Jo, Jooseong Oh, Hyun-Gwan Lee, Hyun-Hee Hong, Sung-Gwon Lee, Seongmin Cheon, Elizabeth M. A. Kern, Soyeong Jin, Sung-Jin Cho, Joong-Ki Park, Chungoo Park

**Correction**

In the final publication of “Draft genome of the sea cucumber *Apostichopus japonicus* and genetic polymorphism among color variants,” by Jihoon Jo et al., the listed accepted date was incorrect, and the article did not include the revised date. The accepted date has been corrected and the revised date added. The corrected manuscript can be found online at https://academic.oup.com/gigascience/article/6/1/1/2865205/Draft-genome-of-the-sea-cucumber-Apostichopus?searchresult=1.

The Publishers regret these errors.

