# Supplementary material for: Draft genome of the sea cucumber Apostichopus japonicus and genetic polymorphism among color variants
Source: Gigascience. 2017 Jan 7;6(1):1–6. doi: 10.1093/gigascience/giw006 (PMC5437941; doi:10.1093/gigascience/giw006)
Supplement: GIGA-D-16-00085_Original_Submission.pdf [file giw006_giga-d-16-00085_original_submission.pdf]

Manuscript

Click here to view linked References

Click here to download Manuscript GigaSience_manuscript_Final_Updated.docx

Data note

Draft genome of the sea cucumber *Apostichopus japonicus* and genetic polymorphism among color variants

Jihoon Jo[a#], Jooseong Oh[a#], Hyun-Gwan Lee[b], Hyun-Hee Hong[a], Sung-Gwon Lee[a], Seongmin Cheon[a], Elizabeth M. A. Kern[c], Soyeong Jin[c], Sung-Jin Cho[d*], Joong-Ki Park[c*], and Chungoo Park[a*]

[a] School of Biological Sciences and Technology, Chonnam National University, Gwangju 61186, Republic of Korea

[b] Marine Ecological Disturbing and Harmful Organisms Research Center, Department of Oceanography, Chonnam National University, Gwangju 61186, Republic of Korea

[c] Division of EcoScience, Ewha Womans University, Seoul 03760, Republic of Korea.

[d] Department of Biology, College of Natural Sciences, Chungbuk National University, Cheongju, Chungbuk 28644, Republic of Korea

[#] These authors equally contributed this work.

*Corresponding Authors.

E-mail addresses:

Chungoo Park, chungoo@jnu.ac.kr. Tel: +82-62-530-1913. Fax: +82-62-530-2199

Joong-Ki Park, jkpark@ewha.ac.kr. Tel: +82-2-3277-5948. Fax: +82-2-3277-2385.

Sung-Jin Cho, sjchobio@chungbuk.ac.kr. Tel: +82-43-261-2294. Fax: +82-43-260-2298.

Abstract

Background: The Japanese sea cucumber (*Apostichopus japonicus* Selenka 1867) is an economically important species as a source of seafood and ingredient in traditional medicine. It is mainly found off the coasts of northeast Asia. Recently, substantial exploitation and widespread biotic disease in *A. japonicus* have generated increasing conservation concern. However, the genomic knowledge base and resources available for researchers to use in managing this natural resource and to establish genetically based breeding systems for sea cucumber aquaculture are still in a nascent stage.

Findings: A total of 312 gigabases (Gb) of raw sequences were generated using the Illumina HiSeq 2000 platform and assembled to a final size of 0.67 Gb which is about 81.7 % of the estimated genome size (0.82 Gb). We observed nucleotide-level heterozygosity within the assembled genome to be 0.986 %. The resulting draft genome assembly comprising 132,607 scaffolds with an N50 value of 10.5 kb contains a total of 21,771 predicted protein-coding genes. We identified 6.6 – 14.5 million heterozygous SNPs in the assembled genome of the three natural color variants (green, red, and black), resulting in an estimated nucleotide diversity of 0.00146.

Conclusions: We report the first draft genome of *A. japonicus* and provide a general overview of the genetic variation in the three major color variants of *A. japonicus*. These data will help provide a comprehensive view of the genetic, physiological, and evolutionary relationships among color variants in *A. japonicus*, and will be invaluable resources for sea cucumber genomic research.

Keywords: Sea cucumber genome, *Apostichopus japonicus*, Color variants, Genetic variation, Population genomics

Data description

Background information on *A. japonicus*

The class Holothuroidea (also known as sea cucumbers) belongs to the phylum Echinodermata and comprises approximately 1,250 recorded species worldwide, including some species that are of commercial and medical value [1, 2]. *Apostichopus japonicus* Selenka 1867 is one of the well-known, commercially important sea cucumber species and occurs in the northwestern Pacific coast including China, Japan, Korea and the Far Eastern seas. This species exhibits a wide array of dorsal/ventral color variants (in particular green, red, and black; Fig 1), which differ in their biological and morphological attributes (e.g., shape of ossicle, habitat preference, spawning period, and polian vesicles) [1, 3]. Recently, overexploitation and the prevalence of biotic disease (viral infections) in sea cucumber aquaculture have generated increasing conservation concern [4, 5]. However, the genomic knowledge base and resources available to researchers for use in managing this natural resource or establishing genetically based breeding systems are still in a nascent stage [6].

Sample collection and genomic DNA extraction

Specimens of the three color *A. japonicus* variants (green, red, and black) were collected from same geographical location (GPS data: 34.1 N, 127.18E, Geomun-do, Yeosu, Republic of Korea). Genomic DNA of each color variant was extracted manually from body wall tissues of single male specimens. Briefly, we ground the tissues to fine powder using mortar and pestle with liquid nitrogen freezing. Tissue powders were digested for 1 hour at 65 ^{o}C in CTAB (Cetyltrimethylammonium bromide) lysis buffer (2% CTAB, 1.4 M NaCl, 20 mM EDTA, 100 mM Tris-HCl, and pH 8.0), followed by

Phenol/Chloroform extraction and ethanol precipitation.

Sequencing and quality control

Using the standard protocol provided by Illumina (San Diego, USA), we constructed both short-insert (180 and 400 bp) and long-insert (2 kb) libraries for 2 x 101 bp paired-end reads, which were sequenced using a HiSeq 2000 instrument. For the green color variant, a total of 225 Gb of raw data was generated from all three libraries. In the case of the red and black color variants, 40 and 47 Gb of raw reads, respectively, were produced by 400 bp short-insert library. The raw reads were preprocessed using Trimmomatic v0.33 [7] and Trim Galore [8], in which reads containing adapter sequences, poly-N sequences, or low quality bases (below a mean Phred score of 20) were removed. To correct for errors in the raw sequences, we used ALLPATHS-LG v52488 [9]. Approximately 208, 39, and 42 billion clean reads were obtained for green, red, and black color variant samples, respectively (Table 1). The *A. japonicus* genome size was estimated to be approximately 0.9 Gb based on k-mer measurement (Fig 2), which is fully consistent with genome size measured by flow cytometry (~ 0.82 Gb) [10]. Based on this estimation, the clean sequence reads correspond to about 356-fold coverage of the *A. japonicus* genome.

Assembly

For whole-genome assembly, we used reads only from green color variant libraries and employed Platanus v1.2.4 [11], which is well suited for high-throughput short reads and heterozygous diploid genomes. Briefly, error corrected paired-end (insert size: 180 bp and 400 bp) reads were input for contig assembly. Next, all cleaned

paired-end (insert size: 180 bp and 400 bp) and mate-paired (insert size: two 2 kb samples) reads were mapped onto the contigs for scaffold building and were utilized for gap filling (any nucleotide represented by “N” in scaffolds). After gap filling by Platanus, the gaps that still remained in the resulting scaffolds were closed using GapCloser (a module of SOAPdenovo2 [12]). The final genome assembly was 0.67 Gb in total length, which is about 81.7 % of the estimated genome size by flow cytometry (0.82 Gb) [10], and is composed of 132,607 scaffolds (> 1 kb) with an N50 value of 10.5 kb (Table 2). We assessed the completeness of the assembly using CEGMA v2.4.010312 [13] and BUSCO v1.22 [14]. 73.4% of the core eukaryotic genes (based on the 248 core essential genes) and 60.7% of the metazoan single-copy orthologs (based on the 843 genes), respectively were identifiable in the genome. Because assembling highly heterozygous genomes is a major challenge in *de novo* genome sequencing, we further sought to explore whether there are other assemblers that could produce better genome assembly statistics. We applied two popular genome assemblers, SOAPdenovo2 2.04-r240 [12] and ALLPATHS-LG v52488 [9], and as expected [11], the Platanus assembler was superior to the others (Table S1).

Annotation

To identify genomic repeat elements in the *A. japonicus* genome assembly, we ran RepeatMasker (version 4.0.6) [15] using the Repbase TE library (release 20150807) [16] and the *de novo* repeat library constructed by RepeatModeler (version 1.0.8) [17]. Approximately 27.2% of the *A. japonicus* genome was identified as interspersed repeats.

Protein-coding genes were predicted using four steps. First, *ab initio* gene prediction was performed with trained AUGUSTUS v3.2.1 [18] using hints from

splicing alignment of transcripts to the repeat-masked assembled genome with BLAT [19] and PASA v2.0.2 [20]. To obtain high quality spliced alignments of expressed transcript sequences for the AUGUSTUS training set, we collected RNA-seq data from our previous [21] and other transcriptome [22] studies, and assembled reads from the RNA-seq dataset using Trinity v2.1.1 [23]. Second, for homology-based gene prediction, homologous proteins in other species (from UniProt [24]) were mapped to the repeat-masked assembled genome using tBLASTn [25] with an E-value ≤ 1 x 10^{-5}. The aligned sequences were predicted using GeneWise v2.4.0 [26] to search for precise spliced alignment and gene structures. Third, for homology-based gene prediction with transcriptome evidence, existing RNA-seq reads [19, 21] were mapped to the repeat-masked assembled genome using TopHat v2.1.0 [27], and gene models were built using Cufflinks v2.2.1 [28]. Finally, the resulting gene sets from each approach were integrated into a comprehensive and non-redundant consensus gene set. We predicted a total of 21,771 (≥ 50 amino acids) genes in the assembled *A. japonicus* genome including 101,776 exons (average 4.67 exons per gene), and an average gene size of 5,402 nucleotides (average transcript size of 982 nucleotides) (Table. 2).

Genetic polymorphism among natural color variants

To provide a general overview of the total genetic variation in the species, we realigned reads from the green color variant to the assembled genome using BWA v0.7.13 [29]. Picard v1.141 (http://picard.sourceforge.net/) was used to mark and remove duplicates. Before SNP and small indel calling, we realigned reads with indels using GATK RealignerTargetCreator and IndelRealigner v3.5 [30] to avoid misalignment around indels. Next, GATK Haplotypecaller was used to call SNPs and

indels from the resulting sequences. In this study, we observed nucleotide-level heterozygosity within the assembled genome to be 0.986 %; namely, we identified a total of 6,550,122 SNPs at the assembled genome, for a heterozygous SNP rate of 0.00986 per site. This high rate of nucleotide polymorphism is not uncommon in marine invertebrates and also have found in the sea urchin genome (~1%; at least one SNP per 100 bases) [31], which belongs to the same phylum.

To measure nucleotide diversity in *A. japonicus*, the aforementioned analyses were repeated for red and black color variants separately, and VCFtools v0.1.14 [32] with sliding window analysis (bin 10 kb, step 1 kb) was used to calculate nucleotide diversity. We identified 6.6 – 14.5 million heterozygous SNPs (1.7 – 3.7 million small indels) in the assembled genome from the three natural color variants (Table 3), resulting in an estimated nucleotide diversity of 0.00146.

In summary, we report the first draft genome of *A. japonicus* and provide a general overview of the genetic variation in its three color variants (green, red, and black). These data will help elucidate the genetic, physiological, and evolutionary relationships among different color variants in *A. japonicus* and will be invaluable resources for sea cucumber genomic research.

Availability of supporting data

The raw dataset of all *Apostichopus japonicus* genome libraries was deposited in the NCBI database with BioProject accession number PRJNA335936 and SRA accession number SRP082485. The additional dataset associated with genome annotation was deposited in GigaDB. The RNA-seq datasets used in this study were downloaded from the ENA database with accession number PRJEB12167 and the NCBI

database with SRA accession number SRA046386.

Abbreviations

bp: base pairs; kb: kilobases; Gb: Gigabases; TE: Transposable element; RNA-seq: High-throughput messenger RNA sequencing; SNP: Single nucleotide polymorphism; Indel: Insertion and deletion.

Competing interests

The authors declare that they have no competing interests.

Authors' contributions

CP designed the study; CP, JKP, SJC contributed to the project coordination; JJ, HGL, HHH, and SJ collected the samples and extracted the genomic DNA; CP, JO, SGL, and SC conducted the genome analyses; CP, JKP, JJ and EK wrote the paper; All authors read and approved the final manuscript.

Acknowledgements

This work was supported by research grants from the Marine Biotechnology Program (PJT200620, Genome Analysis of Marine Organisms and Development of Functional Applications) funded by the Ministry of Oceans and Fisheries of the Republic of Korea to CP, JKP, SJC and from the Basic Science Research Program through the National Research Foundation of Korea (NRF) funded by the Ministry of Science, ICT & Future Planning (NRF-2015R1C1A1A02036896) to CP.

Author details

[a] School of Biological Sciences and Technology, Chonnam National University, Gwangju 61186, Republic of Korea. [b] Marine Ecological Disturbing and Harmful Organisms Research Center, Department of Oceanography, Chonnam National University, Gwangju 61186, Republic of Korea. [c] Division of EcoScience, Ewha Womans University, Seoul 03760, Republic of Korea. [d] Department of Biology, College of Natural Sciences, Chungbuk National University, Cheongju, Chungbuk 28644, Republic of Korea.

References

1. Choe S, Oshima, Y. On the morphological and ecological differences between two commercial forms, “Green” and “Red”, of the Japanese common sea cucumber, Stichopus japonicus Selenka. Nippon Suisan Gakkaishi. 1961;27:97–105.

2. Kanno M, Kijima, A. Quantitative and qualitative evaluation on the color variation of the Japanese sea cucumber Stichopus japonicus. Suisanzoshoku. 2002;50:63–9.

3. Hongsheng Yang J-FH, Annie Mercier. The Sea Cucumber Apostichopus japonicus: History, Biology and Aquaculture. Academic Press; 2015.

4. Bordbar S, Anwar F, Saari N. High-value components and bioactives from sea cucumbers for functional foods--a review. Mar Drugs. 2011;9(10):1761-805. doi:10.3390/md9101761.

5. Purcell SW. Value, market preferences and trade of Beche-de-mer from Pacific Island sea cucumbers. PloS one. 2014;9(4):e95075. doi:10.1371/journal.pone.0095075.

6. Long KA, Nossa CW, Sewell MA, Putnam NH, Ryan JF. Low coverage sequencing of three echinoderm genomes: the brittle star Ophionereis fasciata, the sea star Patiriella regularis, and the sea cucumber Australostichopus mollis. Gigascience. 2016;5:20. doi:10.1186/s13742-016-0125-6.

7. Bolger AM, Lohse M, Usadel B. Trimmomatic: a flexible trimmer for Illumina sequence data. Bioinformatics. 2014;30(15):2114-20. doi:10.1093/bioinformatics/btu170.

8. Krueger F. Trim Galore!: A wrapper tool around Cutadapt and FastQC to consistently apply quality and adapter trimming to FastQ files. 2015. http://www.bioinformatics.babraham.ac.uk/projects/trim_galore/.

9. Gnerre S, Maccallum I, Przybylski D, Ribeiro FJ, Burton JN, Walker BJ et al. High-quality draft assemblies of mammalian genomes from massively parallel sequence data. Proceedings of the National Academy of Sciences of the United States of America. 2011;108(4):1513-8. doi:10.1073/pnas.1017351108.

10. LIU Jin ZX-j, SU Lin, LIU Shi-lin, RU Shao-guo, YANG Hong-sheng. Genome size determination of sea cucumber (Apostichopus japonicus). JOURNAL OF FISHERIES OF CHINA. 2012;Vol.36, No.5. doi:10.3724/SP.J.1231.2012.27753.

11. Kajitani R, Toshimoto K, Noguchi H, Toyoda A, Ogura Y, Okuno M et al. Efficient de novo assembly of highly heterozygous genomes from whole-genome shotgun short reads. Genome research. 2014;24(8):1384-95. doi:10.1101/gr.170720.113.

12. Luo R, Liu B, Xie Y, Li Z, Huang W, Yuan J et al. SOAPdenovo2: an empirically improved memory-efficient short-read de novo assembler. Gigascience. 2012;1(1):18. doi:10.1186/2047-217X-1-18.

13. Parra G, Bradnam K, Korf I. CEGMA: a pipeline to accurately annotate core genes in eukaryotic genomes. Bioinformatics. 2007;23(9):1061-7. doi:10.1093/bioinformatics/btm071.

14. Simao FA, Waterhouse RM, Ioannidis P, Kriventseva EV, Zdobnov EM. BUSCO: assessing

genome assembly and annotation completeness with single-copy orthologs. Bioinformatics. 2015;31(19):3210-2. doi:10.1093/bioinformatics/btv351.

15. Smit AFA, Hubley R, Green P. RepeatMasker 4.0.6. 2015. http://www.repeatmasker.org/.

16. Bao W, Kojima KK, Kohany O. Repbase Update, a database of repetitive elements in eukaryotic genomes. Mob DNA. 2015;6:11. doi:10.1186/s13100-015-0041-9.

17. Smit A, Hubley R. RepeatModeler Open-1.0. 2015. http://www.repeatmasker.org/.

18. Stanke M, Diekhans M, Baertsch R, Haussler D. Using native and syntenically mapped cDNA alignments to improve de novo gene finding. Bioinformatics. 2008;24(5):637-44. doi:10.1093/bioinformatics/btn013.

19. Kent WJ. BLAT--the BLAST-like alignment tool. Genome research. 2002;12(4):656-64. doi:10.1101/gr.229202. Article published online before March 2002.

20. Haas BJ, Delcher AL, Mount SM, Wortman JR, Smith RK, Jr., Hannick LI et al. Improving the Arabidopsis genome annotation using maximal transcript alignment assemblies. Nucleic acids research. 2003;31(19):5654-66.

21. Jo J, Park J, Lee HG, Kern EM, Cheon S, Jin S et al. Comparative transcriptome analysis of three color variants of the sea cucumber Apostichopus japonicus. Marine genomics. 2016. doi:10.1016/j.margen.2016.03.009.

22. Du H, Bao Z, Hou R, Wang S, Su H, Yan J et al. Transcriptome sequencing and characterization for the sea cucumber Apostichopus japonicus (Selenka, 1867). PloS one. 2012;7(3):e33311. doi:10.1371/journal.pone.0033311.

23. Grabherr MG, Haas BJ, Yassour M, Levin JZ, Thompson DA, Amit I et al. Full-length transcriptome assembly from RNA-Seq data without a reference genome. Nat Biotechnol. 2011;29(7):644-52. doi:10.1038/nbt.1883.

24. UniProt C. UniProt: a hub for protein information. Nucleic acids research. 2015;43(Database issue):D204-12. doi:10.1093/nar/gku989.

25. Camacho C, Coulouris G, Avagyan V, Ma N, Papadopoulos J, Bealer K et al. BLAST+: architecture and applications. BMC Bioinformatics. 2009;10:421. doi:10.1186/1471-2105-10-421.

26. Li W, Cowley A, Uludag M, Gur T, McWilliam H, Squizzato S et al. The EMBL-EBI bioinformatics web and programmatic tools framework. Nucleic acids research. 2015;43(W1):W580-4. doi:10.1093/nar/gkv279.

27. Kim D, Pertea G, Trapnell C, Pimentel H, Kelley R, Salzberg SL. TopHat2: accurate alignment of transcriptomes in the presence of insertions, deletions and gene fusions. Genome Biol. 2013;14(4):R36. doi:10.1186/gb-2013-14-4-r36.

28. Trapnell C, Roberts A, Goff L, Pertea G, Kim D, Kelley DR et al. Differential gene and transcript expression analysis of RNA-seq experiments with TopHat and Cufflinks. Nature

protocols. 2012;7(3):562-78. doi:10.1038/nprot.2012.016.
29. Li H, Durbin R. Fast and accurate short read alignment with Burrows-Wheeler transform. Bioinformatics. 2009;25(14):1754-60. doi:10.1093/bioinformatics/btp324.
30. McKenna A, Hanna M, Banks E, Sivachenko A, Cibulskis K, Kernytsky A et al. The Genome Analysis Toolkit: a MapReduce framework for analyzing next-generation DNA sequencing data. Genome research. 2010;20(9):1297-303. doi:10.1101/gr.107524.110.
31. Sea Urchin Genome Sequencing C, Sodergren E, Weinstock GM, Davidson EH, Cameron RA, Gibbs RA et al. The genome of the sea urchin Strongylocentrotus purpuratus. Science. 2006;314(5801):941-52. doi:10.1126/science.1133609.
32. Danecek P, Auton A, Abecasis G, Albers CA, Banks E, DePristo MA et al. The variant call format and VCFtools. Bioinformatics. 2011;27(15):2156-8. doi:10.1093/bioinformatics/btr330.

Table 1. Statistics on total reads of the *Apostichopus japonicus* genome.

Variants	Insertion size (bp)	Total reads* (Raw data)	Total reads* (w/o adaptor)	Total reads* (error corrected)	% error corrected
Green	180	498,608,646	474,117,288	466,062,920	1.70
	400	897,432,174	842,766,704	831,964,242	1.28
	2000 (v1)	293,701,464	270,513,434	268,573,812	0.72
	2000 (v2)	538,359,438	496,446,984	493,387,418	0.62
	Total	2,228,101,722	2,083,844,410	2,059,988,392	1.14
Red	400	397,799,042	394,984,810	383,734,440	2.85
Black	400	460,597,940	423,543,558	416,007,614	1.78

Note: *The length of each read is 101 bp.

Table 2. Statistics on *Apostichopus japonicus* genome assembly

Statistic	Value
Total assembled bases (bp)	664,375,288
Average length of scaffolds (bp)	5,010
Number of scaffolds	132,607
Length of longest scaffold (bp)	131,537
GC content (%)	35.92
N50 (bp)	10,488
Number of genes	21,771
Number of exons per gene	4.67
Average exon length (bp)	209
Number of introns per gene	4.21
Average intron length (bp)	1,048

Table 3. SNP and small indel statistics among three color variants.

Variants	# of heterozygous SNP loci	# of small indel loci
Green	6,550,122	1,662,708
Red	14,509,713	3,681,007
Black	12,627,560	3,198,584

Figure legends

Figure 1. Three color-variants of *Apostichopus japonicus*. (A) Dorsal view of the three color variants. Left to right: red, green, and black. (B) Ventral view of the three color variants. Left to right: red, green, and black.

Figure 2. K-mer distribution of the *Apostichopus japonicus* genome.

Figure 3. Schematic workflow of *Apostichopus japonicus* genome assembly and annotation. The left side represents the genome assembly and the right side represents the transcriptome assembly that was performed in previous publications. To achieve suitable gene prediction, we integrated these two assembly results.

Click here to download Figure Fig_1.tiff

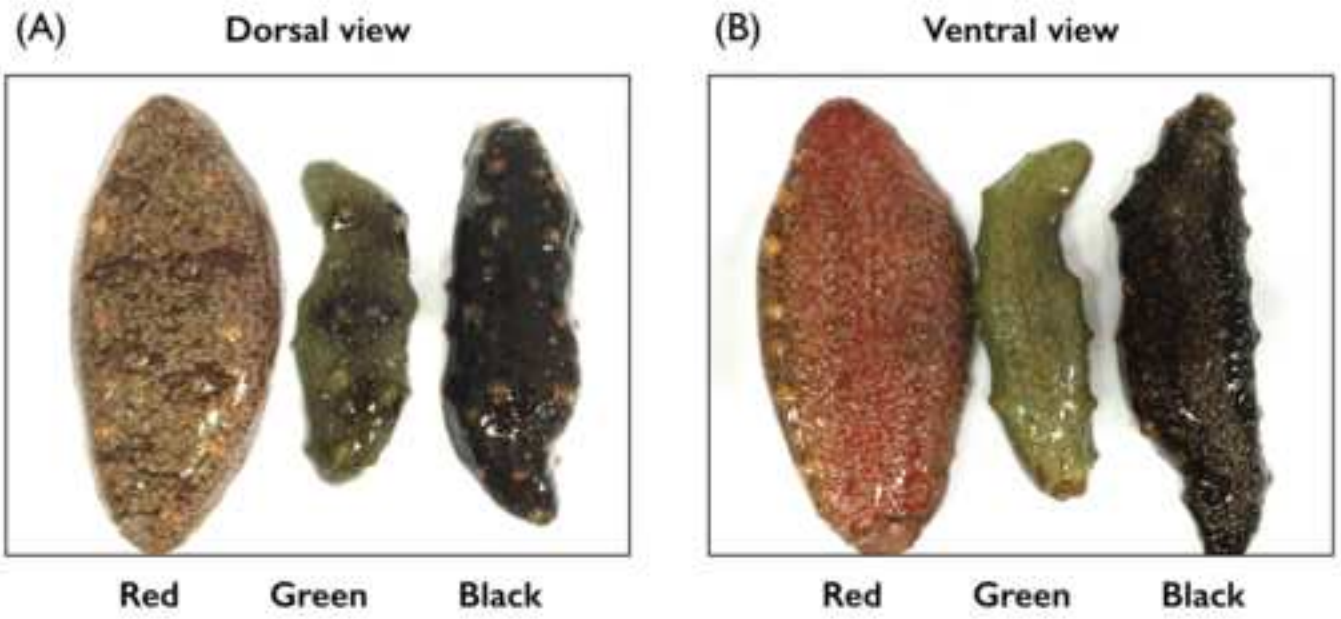

Figure 1

Figure 2

Click here to download Figure Fig_2.tiff

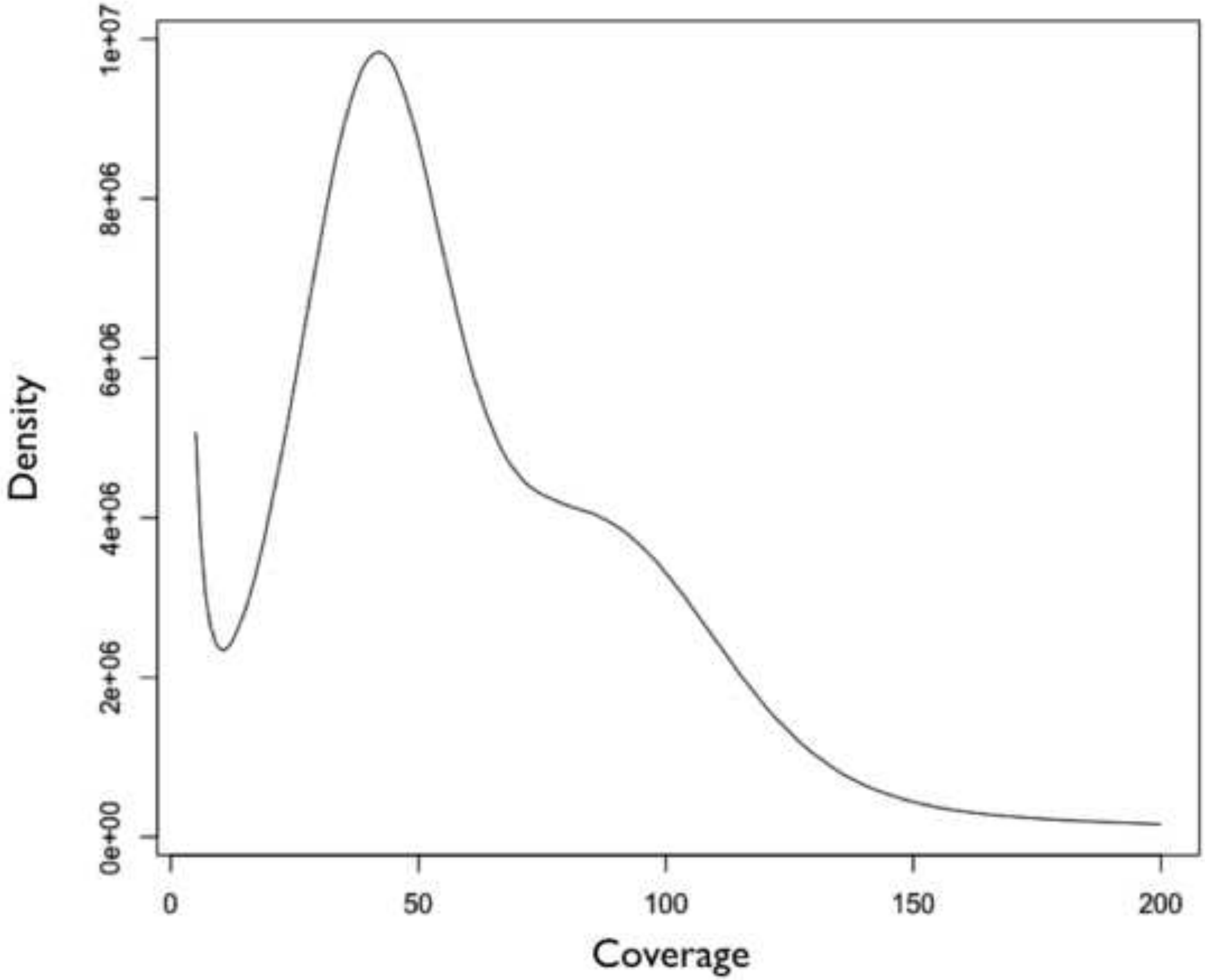

Figure 2

Figure 3

Click here to download Figure Fig_3.tiff

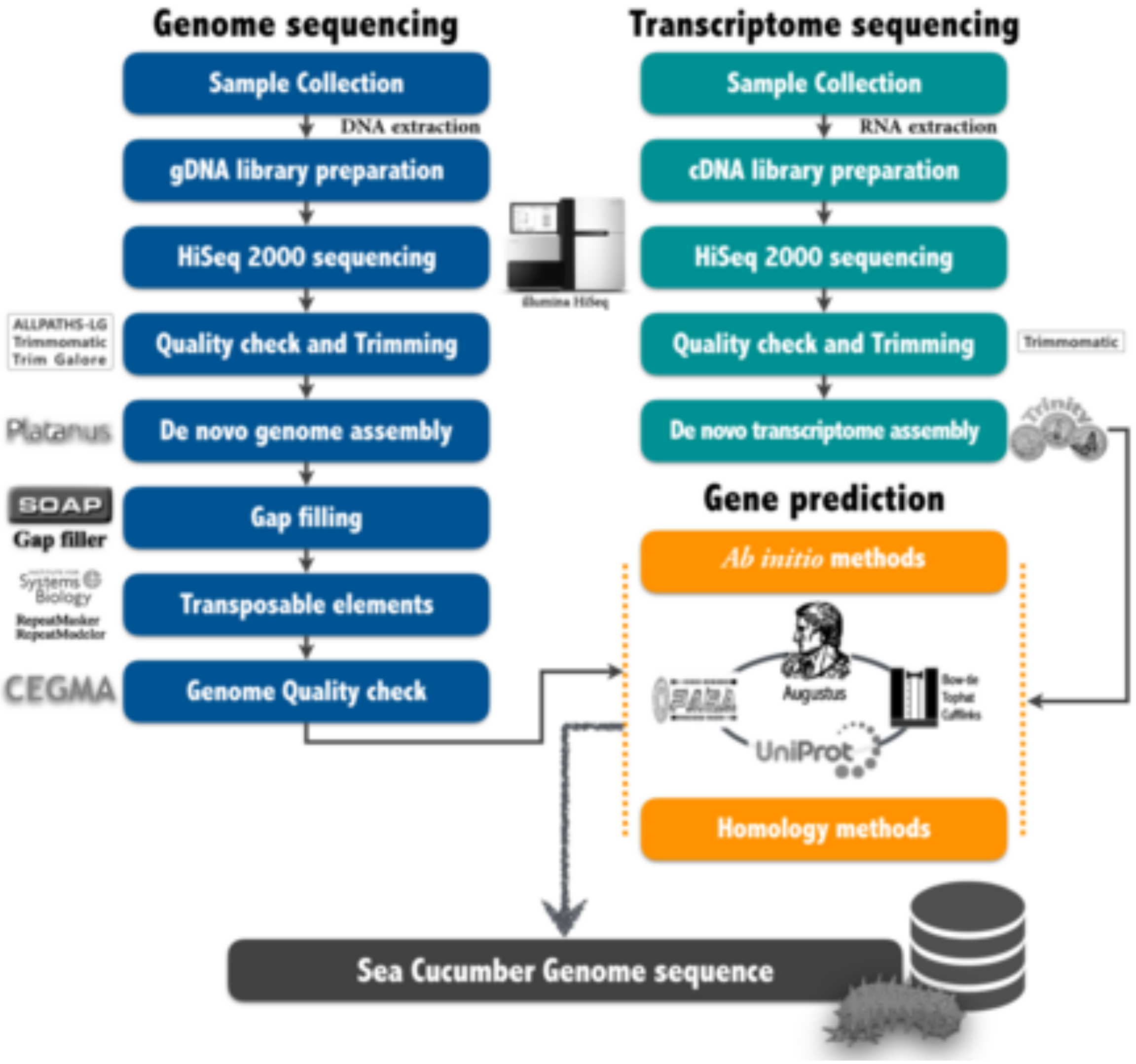

Figure 3

Click here to access/download
Supplementary Material
Table S1.docx

Cover letter Click here to download Personal Cover cover_letter.docx

Sep. 1, 2016.

Dear Editor:

Please consider our manuscript entitled "**Draft genome of the sea cucumber *Apostichopus japonicus* and genetic polymorphism among color variants**" for consideration of publication as a Data note in Gigascience. *Apostichopus japonicus* is one of the well-known, commercially important sea cucumber species and occurs in the northwestern Pacific coast including China, Japan, Korea and the Far Eastern seas. This species exhibits a wide array of dorsal/ventral color variants (in particular green, red, and black), which differ in their biological and morphological attributes. Recently, overexploitation and the prevalence of biotic disease in sea cucumber aquaculture have generated increasing conservation concern. However, the genomic knowledge base and resources available to researchers for use in managing this natural resource or establishing genetically based breeding systems are still in a nascent stage.

We believe that our work is suitable for *Gigascience* for the following two reasons. **First**, We report the first draft genome of *A. japonicas*. A total of 312 gigabases (Gb) of raw sequences were generated using the Illumina HiSeq 2000 platform and assembled to a final size of 0.67 Gb which is about 81.7 % of the estimated genome size (0.82 Gb). We observed nucleotide-level heterozygosity within the assembled genome to be 0.986 %. The resulting draft genome assembly comprising 132,607 scaffolds with an N50 value of 10.5 kb contains a total of 21,771 predicted protein-coding genes. **Second**, we provide a general overview of the genetic variation in the three major color variants of *A. japonicus*. We identified 6.6 – 14.5 million heterozygous SNPs in the assembled genome of the three natural color variants (green, red, and black), resulting in an estimated nucleotide diversity of 0.00146. For the above reasons, we expect that our paper will help provide a comprehensive view of the genetic, physiological, and evolutionary relationships among color variants in *A. japonicus*, and will be invaluable resources for sea cucumber genomic research.

Thank you very much for considering our manuscript.

Sincerely,

Chungoo Park
Assistant Professor of School of Biological Sciences and Technology
Chonnam National University
Gwangju, Republic of Korea, 500-757
Phone: +82-62-530-1913
Email: chungoo@jnu.ac.kr
