# Supplementary material for: Draft genome of the sea cucumber Apostichopus japonicus and genetic polymorphism among color variants
Source: Gigascience. 2017 Jan 7;6(1):1–6. doi: 10.1093/gigascience/giw006 (PMC5437941; doi:10.1093/gigascience/giw006)
Supplement: Response_to_Reveiwer_Comments_Original_Submission.pdf [file giw006_response_to_reveiwer_comments_original_submission.pdf]

November 03, 2016

Dear colleagues at GigaScience,

Thank you very much for handling our manuscript, “Draft genome of the sea cucumber Apostichopus japonicus and genetic polymorphism among color” by Jo et al., which we submitted to GigaScience. The reviewers raised important points that greatly improve our manuscript. Below, we address these points in detail one by one. The reviewers’ comments are in italics. The modifications of the manuscript are shown in red.

Sincerely,

Chungoo Park
Corresponding Author

Editor

Comment 1:
Do you have a picture of the species? We may be able use this to highlight the paper on our homepage, and you may also include a picture of the species as a Figure in your revised manuscript.

Response:
We prepared a picture of the sea cucumber Apostichopus japonicus for the GigaSciecne homepage. We want to use it just as a picture to highlight the paper on the homepage, not as a Figure. Please see the uploaded picture.

Comment 2:
Should you have used any customs scripts or other protocols/materials, please consider providing these via our ftp server.

Response:
In this manuscript, mostly we used well-established bioinformatics tools and pipelines. Because we described all analysis procedures in the manuscript in detail, readers of our manuscript can reproduce all the data without any custom scripts.

Reviewer #1

Comment 1:
The genome assembly is listed as 0.67 Gb. The Assemblathon statistics run on the assembly file

indicates that the total scaffold size 0.66 Gb and the number of scaffolds is 132,497 (both numbers are similar to the numbers reported in the manuscript). But there are 102,722 contigs in scaffolds and 164,958 contigs greater than 1kb, also according to those statistics. Which sequences were used to calculate the completeness statistics - was it with or without the unscaffolded contigs? Did it include the small contigs (20,922 total <1kb)?

Response:
We rechecked the assembly file and it was found that the 0.67 Gb is because 0.664 Gb was incorrected rounded off to the nearest hundredths. Thus, it has been corrected to 0.66 Gb (page 2 and 6).

Next, the reviewer commented that the number of scaffolds generated by the reviewer was slightly different from assembly statistics we present. In the manuscript, we described that the final genome assembly is composed of 132,607 scaffolds with > 1 kb. We found that there is a typo in the range. Actually, we used all scaffolds that are greater than or equal to 1 kb in length. Thus, the "> 1 kb" has been corrected to " 1 kb" (page 6).

We used all scaffolds (which include unscaffolded contigs if they are 1 kb in length) with 1 kb to calculate the completeness statistics.

Comment 2:
Please make it clear the N50 statistics refer to scaffold N50 numbers and also give the contig N50 numbers if the unscaffolded contigs continue to be included in the assembly files.

Response:
We have replaced "N50" with "Scaffold N50" and have added contig N50 with the number of contigs scores in Table 2.

Comment 3:
Also, note if the CEGMA and BUSCO analyses are based upon the assembly as in the files or on the scaffolds only.

Response:
The CEGMA and BUSCO analyses were performed using files we deposited in GigaDB. To be clarified, we have added some words as following (page 6):

"The final genome assembly was 0.66 Gb in total length, which is about 80.5 % of the estimated genome size by flow cytometry (0.82 Gb) [14], and is composed of 132,607 scaffolds and unscaffolded contigs (1 kb) with an N50 value of 10.5 kb (Table 2). We assessed the completeness of the assembly using CEGMA v2.4.010312 [17] and BUSCO v1.22 [18]."

Comment 4:

The genome assembly should be submitted to the International Nucleotide Sequence Databases (DDBJ/NCBI/EMBL).

Response:
We have deposited our genome assembly in NCBI and have now added the GenBank accession number MODV00000000 in manuscript (page 9).

Note that the deposit is confirmed (because we have had the accession number) and now under processing to release our data in public [Figure R1 attached].

Reviewer #2

Comment 1:
A small description (a few sentences) referencing what is known of the genetics/biology of the color morphs used in the project would be useful to the reader.

Response:
We have added the following to the Data description (page 4):

"The red variant is found on rock pebbles and gravel substrate and has higher salinity and temperature tolerance than the other color variants [4, 5]. Green and black variants are found on sandy and muddy bottoms at shallower depths, and the green variant has greater plasticity in thermotolerance than the red variant [6, 7]."

Comment 2:
The authors created other assemblies using different methods that appear less complete (Table S1) but did they analyze these using their core eukaryotic and metazoan gene sets (or with other approaches) to assess potential non-overlap from the alternative assemblies. Could the contribute unique sequence?

Response:
We assessed the completeness of the assemblies from two different methods.
In the genome assemblies from SOAPdenovo and ALLPATHS-LG, only 50 % and 24.6 % of the core eukaryotic genes (based on the 248 core essential genes using CEGMA) were detected, respectively (vs. 73.4% in our final assembly). Using BUSCO, 21.9 % (28.7 %) and 12.1 % (17.2%) of the metazoan (eukaryotoa) single-copy orthologs in the genome assemblies from SOAPdenovo and ALLPATHS-LG, respectively were identified (vs. 60.7% in our final assembly). We have added these results in Table S1.

[Figure R2 attached]
Figure. Results of CEGMA and BUSCO using the assemblies from two different methods

Next, to assess to extent to which unique sequences from alternative assemblies have existed, we aligned all scaffold sequences from alternative assemblies with our final assembled genome sequences. Using e-value < 10-10, 97.7% and 99.1% of scaffolds in SOAPdenovo and ALLPATHS-LG assemblies, respectively, were similar to those in our final assembly.

In summary, our final assembly was superior to the others, consistent with our main argument in the manuscript.

Comment 3:
What transcriptome resources were used in the annotation process and what tissues/ developmental stages did these come from?

Response:
We have added the following to the Data description (page 7):

“we collected RNA-seq data from our previous [25] (from body wall tissue of adult stage specimens) and other transcriptome (from embryo, larva, and juvenile stages [developmental-stage specific]; from gonads, intestines, respiratory trees, and coelomic fluid of adults [tissue-specific]) [26] studies,”
