# Supplementary material for: Draft genome of the sea cucumber Apostichopus japonicus and genetic polymorphism among color variants
Source: Gigascience. 2017 Jan 7;6(1):1–6. doi: 10.1093/gigascience/giw006 (PMC5437941; doi:10.1093/gigascience/giw006)
Supplement: Table S1 [file giw006_supp.docx]

**Table S1. Statistics on *Apostichopus japonicus* genome assembly from three assemblers**

|  | Platanus | SOAPdenovo | ALLPATHS-LG |
| --- | --- | --- | --- |
| Total assembled bases (bp) | 664,375,288 | 895,889,709 | 269,436,422 |
| Average length of scaffolds (bp) | 5,010 | 1,377 | 1,888 |
| Number of scaffolds | 132,607 | 650,693 | 142,609 |
| Scaffold N50 (bp) | 10,488 | 3,195 | 1,918 |
| ^1^CEGMA (%) | 73.4 | 50 | 24.6 |
| ^2^BUSCO (%) | 60.7 | 21.9 | 12.1 |

^1^Percentage of the 248 core eukaryotic genes (CEGs) recovered.

^2^Percentage of the 843 metazoan orthologous genes recovered.
