# Supplementary material for: Draft genome of the sea cucumber Apostichopus japonicus and genetic polymorphism among color variants
Source: Gigascience. 2017 Jan 7;6(1):1–6. doi: 10.1093/gigascience/giw006 (PMC5437941; doi:10.1093/gigascience/giw006)
Supplement: Response_to_Reviewer_Comments_Revision_1.pdf [file giw006_response_to_reviewer_comments_revision_1.pdf]

Author's response to reviews:

Revision version: 2
Date: November 11, 2016

Dear colleagues at GigaScience,

Thank you very much for handling our manuscript, “Draft genome of the sea cucumber Apostichopus japonicus and genetic polymorphism among color” by Jo et al., which we submitted to GigaScience. The reviewers raised important points that greatly improve our manuscript. Below, we address these points in detail one by one. The reviewers’ comments are in italics. The modifications of the manuscript are shown in red.

Sincerely,

Chungoo Park
Corresponding Author

Reviewer

Comment 1:
We are happy to proceed to acceptance of your manuscript, but before going on with the process please include a working accession number (the bioproject PRJ number) in the manuscript.

Response:
In the previous version of our manuscript, we already added the bioproject PRJ number as follow:

“The raw dataset of all Apostichopus japonicus genome libraries and the assembly was deposited in the NCBI database with BioProject accession number PRJNA335936, SRA accession number SRP082485, and GenBank accession number MODV00000000.” (page 9)

Comment 2:
You write: "Next, the reviewer commented that the number of scaffolds generated by the reviewer was slightly different from assembly statistics we present. In the manuscript, we described that the final genome assembly is composed of 132,607 scaffolds with > 1 kb. We found that there is a typo in the range. Actually, we used all scaffolds that are greater than or equal to 1 kb in length. Thus, the “> 1 kb” has been corrected to “ 1 kb” (page 6). "

I'm not sure whether I understand in how far this answers the referee's question - is the difference really caused by also including scaffolds that are exactly 1 kb, in addition to larger ones? I feel this should be more clearly explained in the manuscript ("scaffolds that are greater than or equal to 1 kb").

Response:
Yes, all scaffolds are greater than or equal to 1 kb in length. To clarify it, we modified the sentence (page 6) as follow:
“The final genome assembly was 0.66 Gb in total length, which is about 80.5 % of the estimated genome size by flow cytometry (0.82 Gb) [14], and is composed of 132,607 scaffolds and unscaffolded contigs (that are longer than or equal to 1 kb) with an N50 value of 10.5 kb (Table 2).”

Comment 3:
Thank you also for submitting the beautiful photos. I feel they would be quite suitable to be included as a figure in the manuscript itself, if you have the rights to reproduce them in an open access paper, but that's

totally up to you.

Response:
We replaced our original figure 1 with the picture for homepage. And the legend of figure 1 was modified as follow:

“Figure 1. Three color-variants of Apostichopus japonicus (the green, red, and black variants).”
