# Supplementary material for: Draft genome of the sea cucumber Apostichopus japonicus and genetic polymorphism among color variants
Source: Gigascience. 2017 Jan 7;6(1):1–6. doi: 10.1093/gigascience/giw006 (PMC5437941; doi:10.1093/gigascience/giw006)
Supplement: Reviewer_1_Report_(Original_Submission).pdf [file giw006_reviewer_1_report_(original_submission).pdf]

Reviewer Report

Title: "Draft genome of the sea cucumber Apostichopus japonicus and genetic polymorphism among color variants"

Version: Original Submission **Date:** 9/22/2016

Reviewer name: Kim Worley

Reviewer Comments to Author:

The genome assembly is listed as 0.67 Gb. The Assemblathon statistics run on the assembly file indicates that the total scaffold size 0.66 Gb and the number of scaffolds is 132,497 (both numbers are similar to the numbers reported in the manuscript). But there are 102,722 contigs in scaffolds and 164,958 contigs greater than 1kb, also according to those statistics. Which sequences were used to calculate the completeness statistics - was it with or without the unscaffolded contigs? Did it include the small contigs (20,922 total <1kb)? Please make it clear the N50 statistics refer to scaffold N50 numbers and also give the contig N50 numbers if the unscaffolded contigs continue to be included in the assembly files. Also, note if the CEGMA and BUSCO analyses are based upon the assembly as in the files or on the scaffolds only.The genome assembly should be submitted to the International Nucleotide Sequence Databases (DDBJ/NCBI/EMBL).

Level of Interest

Please indicate how interesting you found the manuscript: An article whose findings are important to those with closely related research interests

Quality of Written English

Please indicate the quality of language in the manuscript: Acceptable

Declaration of Competing Interests

Please complete a declaration of competing interests, considering the following questions:

- Have you in the past five years received reimbursements, fees, funding, or salary from an organisation that may in any way gain or lose financially from the publication of this manuscript, either now or in the future?
- Do you hold any stocks or shares in an organisation that may in any way gain or lose financially from the publication of this manuscript, either now or in the future?
- Do you hold or are you currently applying for any patents relating to the content of the manuscript?
- Have you received reimbursements, fees, funding, or salary from an organization that holds or has applied for patents relating to the content of the manuscript?

- Do you have any other financial competing interests?
- Do you have any non-financial competing interests in relation to this paper?

If you can answer no to all of the above, write 'I declare that I have no competing interests' below. If your reply is yes to any, please give details below.

I declare I have no competing interests.

I agree to the open peer review policy of the journal. I understand that my name will be included on my report to the authors and, if the manuscript is accepted for publication, my named report including any attachments I upload will be posted on the website along with the authors' responses. I agree for my report to be made available under an Open Access Creative Commons CC-BY license (http://creativecommons.org/licenses/by/4.0/). I understand that any comments which I do not wish to be included in my named report can be included as confidential comments to the editors, which will not be published.

I agree to the open peer review policy of the journal
