# Supplementary material for: Draft genome of the sea cucumber Apostichopus japonicus and genetic polymorphism among color variants
Source: Gigascience. 2017 Jan 7;6(1):1–6. doi: 10.1093/gigascience/giw006 (PMC5437941; doi:10.1093/gigascience/giw006)
Supplement: Reviewer_2_Report_(Original_Submission).pdf [file giw006_reviewer_2_report_(original_submission).pdf]

Reviewer Report

Title: "Draft genome of the sea cucumber Apostichopus japonicus and genetic polymorphism among color variants"

Version: Original Submission **Date:** 10/14/2016

Reviewer name: Jonathan Rast

Reviewer Comments to Author: The authors document the production and analysis of a genome assembly from the sea cucumber Apostichopus japonicas. This species has significant commercial value and is the subject of considerable comparative research against the background of other available echinoderm genomes. The manuscript is concise and well-written. It is a useful description consolidating the biological and computational aspects of the genome sequence assembly process and will be a convenient resource for researchers using the sequence.

Below are a few comments for clarifications that may improve the manuscript.

1. A small description (a few sentences) referencing what is known of the genetics/biology of the color morphs used in the project would be useful to the reader.

2. The authors created other assemblies using different methods that appear less complete (Table S1) but did they analyze these using their core eukaryotic and metazoan gene sets (or with other approaches) to assess potential non-overlap from the alternative assemblies. Could the contribute unique sequence?

3. What transcriptome resources were used in the annotation process and what tissues/ developmental stages did these come from?

Click here to enter text.

Level of Interest

Please indicate how interesting you found the manuscript: An article of importance in its field

Quality of Written English

Please indicate the quality of language in the manuscript: Acceptable

Declaration of Competing Interests

Please complete a declaration of competing interests, considering the following questions:

- Have you in the past five years received reimbursements, fees, funding, or salary from an organisation that may in any way gain or lose financially from the publication of this manuscript, either now or in the future?
- Do you hold any stocks or shares in an organisation that may in any way gain or lose financially from the publication of this manuscript, either now or in the future?
- Do you hold or are you currently applying for any patents relating to the content of the manuscript?
- Have you received reimbursements, fees, funding, or salary from an organization that holds or has applied for patents relating to the content of the manuscript?
- Do you have any other financial competing interests?
- Do you have any non-financial competing interests in relation to this paper?

If you can answer no to all of the above, write 'I declare that I have no competing interests' below. If your reply is yes to any, please give details below.

I declare that I have no competing interests

I agree to the open peer review policy of the journal. I understand that my name will be included on my report to the authors and, if the manuscript is accepted for publication, my named report including any attachments I upload will be posted on the website along with the authors' responses. I agree for my report to be made available under an Open Access Creative Commons CC-BY license (http://creativecommons.org/licenses/by/4.0/). I understand that any comments which I do not wish to be included in my named report can be included as confidential comments to the editors, which will not be published.

I agree to the open peer review policy of the journal
